# The Link between Job Demands, Burnout, and the Self-Undermining of Healthcare Employees during the COVID-19 Pandemic: An Underestimated Threat?

**DOI:** 10.3390/healthcare10081408

**Published:** 2022-07-27

**Authors:** Florinda Golu, Adriana Cotel, Nicu Ionel Sava, Bogdan Oprea, Sergiu Condrea

**Affiliations:** Department of Psychology, University of Bucharest, 050663 București, Romania; florinda.golu@fpse.unibuc.ro (F.G.); adriana.cotel@yahoo.com (A.C.); savanicuionel.fpse.unibuc@gmail.com (N.I.S.); sergiucondrea12@gmail.com (S.C.)

**Keywords:** job demands, burnout, self-undermining, COVID-19, healthcare

## Abstract

Previous studies have shown an association between job demands and burnout in medical staff during the pandemic. However, these studies have ignored the possibility of loss cycle occurrence during the crisis. In order to address this gap, the aim of this study was to test the mediating role of burnout in the positive relationship between job demands and the self-undermining of healthcare employees during the COVID-19 pandemic. Self-undermining represents the set of behaviors that generate obstacles and diminish performance (e.g., careless mistakes, generating interpersonal conflicts, poor communication), and it has been suggested that this variable could explain the loss cycle between demands and burnout (the phenomenon in which demands increase burnout, which in turn leads to even greater demands). A total of 523 healthcare workers from two Romanian hospitals (e.g., physicians, nurses, stretcher-bearers) completed a self-report questionnaire during the COVID-19 outbreak that included three job demands, burnout, and self-undermining. Burnout mediated the positive relationship between two demands (work–family conflict; lack of equipment and supplies) and self-undermining. These results may provide a preliminary indication for the existence of loss cycles, supporting the assumptions of the job demands-resources theory. Moreover, the results highlight the risk of loss cycle occurrences for healthcare employees during outbreaks.

## 1. Introduction

The coronavirus disease (COVID-19) pandemic is a major challenge for healthcare workers, who have reported high levels of burnout (a work-related syndrome that includes exhaustion, cynicism, and reduced professional efficacy [1]) during this difficult situation [2]. A global survey indicated that some of the factors that are associated with high levels of healthcare employees’ burnout during the pandemic are being pushed beyond training, high time pressure, making life-or-death prioritizing decisions, and a lack of organizational support [3]. These findings are in line with over 20 years of research [4] indicating that burnout occurs in work environments characterized by a high level of job demands (requirements that involve sustained physical, mental, or emotional effort on the part of employees [5]). Although previous studies have highlighted the association between job demands and burnout among healthcare workers during the pandemic, these studies may have underestimated the negative impact of demands and exhaustion on healthcare worker behavior.

Recent studies indicate that the link between job demands and burnout is bidirectional, meaning that job demands predict the subsequent level of burnout, while burnout leads to a higher level of subsequent demands [6]. The phenomenon in which these relationships are circular (i.e., demands increase burnout, which in turn leads to even greater demands) is referred to as a “loss cycle”, and researchers suggest it may be explained by self-undermining behaviors [7]. Self-undermining is described as a set of behaviors that generate obstacles and diminish performance [7], such as careless mistakes, generating interpersonal conflicts, or poor communication [8]. The loss cycle appears when the job demands translate into burnout, which in turn leads to self-undermining behaviors that increase the already existing high level of job demands [9,10]. In other words, when employees have to deal with overwhelming demands, they are more likely to experience high levels of exhaustion, cynicism, and reduced professional efficacy. When exhausted, employees tend to become involved in maladaptive self-regulation behaviors, creating conflicts, making errors, and communicating inefficiently. These self-undermining behaviors generate hindrance job demands (e.g., role ambiguity, emotional demands, and hassles), which could further increase exhaustion. We expect the same loss cycle for healthcare workers during the COVID-19 pandemic. In line with the loss cycle hypothesis, the aim of this study was to test the mediating role of burnout in the positive link between job demands and the self-undermining of healthcare employees during the COVID-19 pandemic.

This study considered employees in the Romanian healthcare system. Romanian medical staff were exposed to high job demands due to inadequate healthcare system infrastructure, which was generally built during communism [11]. The lack of supplies and necessary equipment, citizens’ lack of trust in the health system, and citizens who did not follow the recommendations of the authorities increased the infection rate [11]. From a theoretical perspective, this study contributes to understanding the impact of job demands on healthcare workers during a pandemic by highlighting unconsidered consequences (i.e., self-undermining behaviors). In addition, this study may provide preliminary evidence for the possibility of the existence of loss cycles in healthcare workers during crises. Given that burnout is negatively associated with the quality of care and the safety of patients [12], this study also has important practical implications. The high prevalence of burnout in medical staff and its negative consequences could be impediments to effectively coping with a health crisis. In order for the authorities to take sustainable measures in such situations, it is important that they understand the dynamics between healthcare employees’ job demands and burnout and its behavioral consequences during the pandemic, and to identify solutions to reduce the negative effects.

### 1.1. Job Demands and Burnout

The interplay between job demands and burnout is explained by the job demands-resources (JD-R) theory [10]. According to the theory, job demands initiate a health impairment process in which employees must exert physical, mental, and emotional effort in order to handle workplace requests or to cope with job hindrances (e.g., role ambiguity, interpersonal conflicts). If exposure to these stressors is prolonged or if the demands are overwhelming, employees will deplete their energy and available resources and will be more likely to feel exhausted. Meta-analytical findings support this assumption of the theory, showing that there is a positive association between job demands and burnout [4]. This relationship was also highlighted in the context of the pandemic among employees from the medical field [3], indicating that this theory can be extended to healthcare crises.

Previous studies have already identified a number of pandemic-related demands that are positively associated with burnout in medical staff, such as a high risk of infection, time pressure, making life-or-death decisions, and heavy workloads [3,13]. For the present study, we selected three job demands due to their relevance to the medical field during the pandemic: work–family conflict, lack of comfort with working conditions, and lack of equipment and supplies. Work–family conflict arises when responsibilities in one area of life (work or family) make it difficult to meet responsibilities in another area (family or work); for example, when employees work overtime, they no longer have the time to properly take care of their children [14]. Due to the increasing demands during the pandemic, high workloads may require the extension of the work schedule. Hence, employees in the medical field are expected to spend less time at home due to these additional requests. During the pandemic, physicians and nurses may perceive that workplace requirements affect their personal and family life, and that time spent at work makes it difficult for them to fulfil family responsibilities. These additional demands caused by the pandemic may lead to canceling plans for family activities and to reduced leisure time. All these changes can cause stress and burnout. Therefore, we expected a positive association between work–family conflict and burnout in healthcare workers during the pandemic.

Given the increase in the number of patients and the high volume of work, we expected an increase in the perception of unfavorable working conditions. These conditions mainly refer to the excessive physical or mental effort that employees in the medical field have to make [15]. As the theoretical model argues [10], when the workload is high, the effort required to perform the tasks is also expected to be high. Thus, the burnout of healthcare employees could increase. We expected a positive association between the lack of comfort with working conditions and the burnout of healthcare employees during the COVID-19 pandemic.

Finally, another demand we considered, based on the existing literature, is the lack of equipment and supplies. This refers to the fact that the necessary equipment is missing or poorly maintained and that the supplies needed for the work are not available [15]. Given the new demands that employees in healthcare have to face during the pandemic, the need for equipment may not be met in some hospitals, or supplies may run out too soon; therefore, these issues can become sources of stress. The perception of a lack of necessary equipment and supplies was expected to be associated with a higher level of burnout among employees in the medical field during the COVID-19 pandemic.

**Hypothesis** **1.** *Healthcare employees’ burnout is positively associated with job demands during the COVID-19 pandemic*.

### 1.2. Self-Undermining

Bakker and Costa [7] extended the JD-R model by including the reactions that employees may have when they face exhaustion. In order to deal with burnout, employees may use adaptive or maladaptive self-regulation strategies [8]. According to Bakker and de Vries [8], the adaptive strategies include recovery (through leisure activities such as hobbies, sports, and socializing) and job crafting (proactively changing job characteristics in order to create a balance between resources and demands). However, the exceptional situation during the pandemic could prevent employees from engaging in such adaptive behaviors. The high number of patients in need of emergency medical care and the rapid changes in crisis management strategies could reduce the leisure time of health workers. Additionally, standard working procedures and rigid working conditions during the pandemic could affect employees’ autonomy and their ability to engage in job crafting behaviors. Given that these employees may not be able to choose adaptive depletion management strategies, their level of exhaustion could increase and lead to dysfunctional behaviors.

One form of maladaptive regulation response is self-undermining [7], representing behaviors that occur when employees no longer have the energy to regulate their emotions and cognitive functions, which leads to mistakes, conflicts, and impaired performance [8]. Failure in cognitive and emotional self-regulation may lead to mistakes in tasks, the uncontrolled expression of negative emotions, and interpersonal conflicts. Indeed, burnout can be detrimental to cognitive functioning, attention, and the inhibition of undesirable behaviors [16]. In addition, it can lead to inadequate emotional regulation strategies, which harm social interactions at work [17]. These behaviors can generate a loss cycle.

Previous studies have provided clues to the existence of loss cycles, although not in crisis contexts. For example, in a longitudinal study [18], the researchers found reciprocal relationships between work–home interference, work pressure (two job demands), and exhaustion, meaning that work pressure and work–family interference predicted exhaustion later, but exhaustion also led to a higher level of the two demands later. In another study [19], the initial burnout of employees led to a higher level of subsequent burnout, the relationship being explained by the increase in work overload. These mutual associations suggest the existence of a loss cycle. Self-undermining is supposed to be the explanatory mechanism for these two-way relationships. We expected to find similar preliminary clues for the existence of loss cycles during the pandemic. In line with the theoretical assumption, we expected burnout to mediate the positive link between job demands and self-undermining.

**Hypothesis** **2.** *Healthcare employees’ burnout mediates the positive relationship between job demands and self-undermining during the COVID-19 pandemic*.

## 2. Materials and Methods

### 2.1. Participants and Procedure

As part of a larger research project that investigated the antecedents and outcomes of burnout in medical staff during the pandemic, two hospitals in Romania were contacted in order to distribute the study questionnaire to their employees. The leaders of the hospitals were informed about the purpose of the study and were asked for permission to collect data. The managers or decision makers of the two hospitals received the link for the online version of the questionnaire or envelopes with the paper-and-pencil questionnaires. Participants who completed the printed questionnaire could then seal the envelope. Only the researchers had access to the participants’ answers. All ethical norms in research were followed. The data were collected during the COVID-19 pandemic, from March to June 2020. A total of 544 employees completed the questionnaire. Twenty-one responses were eliminated due to missing values. For the remaining 523 responses, 99 were from men (18.1%) and 424 were from women (81.1%), who had a mean age of 42.86 years (SD = 9.43). Of these 523 participants, 149 (28.5%) were physicians, 349 (66.7%) were nurses, and 25 (4.8%) had other occupations in the hospitals (e.g., stretcher-bearers). The job tenure of the healthcare employees was as follows: under 1 year (5%), between 1 and 3 years (8%), between 3 and 5 years (14.9%), between 5 and 10 years (7.1%), and over 10 years (65%).

### 2.2. Measures

Job demands were measured with the Job Demands in Nursing Scale [15], which uses a scale from 1 (strongly disagree) to 5 (strongly agree). Lack of comfort with working conditions was measured with 4 items (e.g., “I am comfortable with the amount of physical effort required for my work”), and lack of equipment and supplies was measured with 3 items (e.g., “The equipment that I need to do my work is readily available”). Finally, work–family conflict was measured with the Work–Family Conflict Scale [14], composed of 5 items on a 7-point (strongly disagree–strongly agree) response scale (e.g., “My job produces strain that makes it difficult to fulfill family duties”).

Burnout was measured using the Maslach Burnout Inventory—General Survey [20,21]. The 16 items of the scale measure three components of burnout on a 7-point Likert scale, from 0 (never) to 6 (every day): exhaustion (5 items, e.g., “I feel emotionally drained from my work”), cynicism (5 items, e.g., “I feel used up at the end of the workday”), and professional inefficacy (6 items, e.g., “Working all day is really a strain for me”).

Self-undermining was measured using the Self-Undermining Scale [10]. This scale contains 6 items on a 5-point Likert scale, from 1 (never) to 5 (very often) (e.g., “I create a backlog in my tasks”).

## 3. Results

### 3.1. Measurement Model

By using the lavaan package [22] in R 3.6.1 [23], we conducted a confirmatory actor analysis in order to test the factor structure of the measures. The goodness of fit of the measurement model was assessed with the following indices: root mean square error of approximation (RMSEA) < 0.06 [24,25], comparative fit index (CFI) > 0.90, Tucker–Lewis index (TLI) > 0.90 [26], and standardized root mean square residuals (SRMS) < 0.08 [24,25], which indicate an adequate fit. In our five-factor measurement model, every job demand was loaded by its specific items, self-undermining was loaded by its six specific items, and burnout was a second-order factor loaded by its three specific dimensions (i.e., exhaustion, cynicism, and professional inefficacy), each loaded by its specific items. A few error correlations within items suggested by the modification indices were allowed. The fit indices indicated an acceptable fit of the model (χ^2^ = 1219.20, df = 511, RMSEA = 0.05, CFI = 0.91, TLI = 0.90, SRMR = 0.07).

### 3.2. Descriptive Statistics and Correlations between the Variables

Table 1 shows the means, standard deviations, reliabilities, and zero-order correlations among the variables included in the study. All job demands were positively related to burnout. Except for lack of comfort with working conditions, all demands positively correlated with self-undermining.

### 3.3. Mediation Analysis

**Figure 1:**
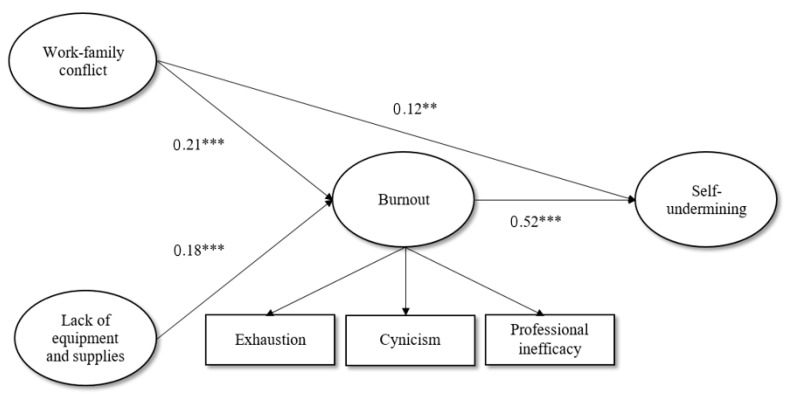
Standardized estimates for the relationships between job demands, burnout, and self-undermining. Note: ** *p* < 0.01; *** *p* < 0.001.

A path analysis with latent variables was conducted using the lavaan package for r in order to test the mediating role of burnout in the positive relationship between job demands and self-undermining. Direct and indirect paths from two job demands (work–family conflict, lack of equipment and supplies) to self-undermining through burnout were found. Lack of comfort with working conditions was excluded from the mediation model because the zero-order correlation with self-undermining was statistically insignificant. The fit indices for the mediation model indicated an acceptable fit (χ^2^ = 903.767, df = 393, RMSEA = 0.05, CFI = 0.93, TLI = 0.92, SRMR = 0.06). Work–family conflict had a total effect of b = 0.23, *p* < 0.001, an indirect effect of b = 0.11, *p* < 0.001, and a direct effect of b = 0.12, *p* < 0.001; lack of equipment and supplies had a total effect of b = 0.15, *p* < 0.01, an indirect effect of b = 0.10, *p* < 0.01, and an insignificant direct effect of b = 0.05, *p* > 0.05. Burnout partially mediated the link between work–family conflict and self-undermining and totally mediated the link between lack of equipment and supplies and self-undermining. The standardized effects are presented in Figure 1.

## 4. Discussion

Previous studies have highlighted the impact that the COVID-19 pandemic had on the well-being of employees in the medical field. During the outbreak, the prevalence of burnout among healthcare employees reached values of 51% [27]. This prevalence can be explained by the demands faced by medical staff in such situations, including the high risk of infection, inadequate resources, heavy workloads, and work pressure [13]. Although these studies have pointed out the negative impact of the demands during the pandemic, this negative impact may be underestimated by the existing literature. Given that the relationship between job demands and burnout is bidirectional, the pandemic could lead to a loss cycle (a situation in which demands increase burnout and burnout, in turn, leads to even greater demands). This two-way link may be explained by employees’ self-undermining behaviors, such as poor communication, interpersonal conflicts, and careless mistakes [7]. According to the loss cycle hypothesis, when confronted with overwhelming demands, employees experience a high level of burnout. Due to exhaustion, employees lose their ability to adjust cognitively and emotionally, which causes even higher demands. In previous studies, the possible existence of this two-way link between demands and burnout was not investigated during a health crisis, such as a pandemic.

We intended to cover this gap by testing the mediating role of burnout in the positive relationship between job demands and the self-undermining of healthcare employees during the COVID-19 pandemic. Positive associations were found for almost all variables. The only variable that was not associated with self-undermining was a lack of comfort with working conditions. Our results are in line with previous studies, in which self-undermining was positively related to exhaustion [10] and perceptions of job demands, workload, and emotional load [28]. Additionally, burnout mediated the relationship between two demands (work–family conflict and lack of equipment and supplies) and self-undermining behaviors. Although our study was cross-sectional, the results can be seen as an initial indication for the existence of a loss cycle.

### 4.1. Theoretical and Practical Implications

From a theoretical perspective, these results may provide a preliminary indication for the existence of loss cycles, as the job demands-resources theory suggests [7]. Previous research already indicated that job demands are strong predictors of employee burnout [4], including during the pandemic for employees in the medical field [3]. Additionally, existing studies support the reciprocal relationship between job demands and burnout. However, the explanation for this two-way relationship has not been tested thus far. The job demands-resources theory suggests [7] that the interplay between demands and burnout can be explained by self-undermining behaviors. In line with the loss cycle hypothesis, job demands should lead to a higher level of burnout, which in turn should lead to self-undermining behaviors that further increase demands. Our findings indicate that burnout mediates the positive link between job demands and self-undermining. This result can be seen as a preliminary indication for the existence of a loss cycle.

From a practical point of view, our results highlight the risk of loss cycle occurrences for healthcare employees during outbreaks. According to the job demands-resources theory [5], practitioners may implement interventions to increase employees’ personal or job resources in order to diminish the relationship between demands and both burnout and self-undermining. Meta-analytical studies have identified such interventions that can reduce burnout or increase engagement, and that could be tested during pandemics: relaxation techniques, cognitive behavioral therapy, work-related knowledge and skills development [29], and job crafting interventions [30].

### 4.2. Limitations and Future Directions

This study has a number of limitations. The research was cross-sectional, and the measurements were self-reported; therefore, we cannot draw causal conclusions and cannot control for reporting biases. Future studies could test this suggested loss cycle with multiple waves and various sources of measurement. Indeed, previous studies that explored the two-way link between demands and burnout were longitudinal [18,19]. In these studies, job demands and burnout were measured at multiple time points; therefore, the researchers were able to show that demands predict the subsequent level of burnout and vice versa. However, self-undermining, as a possible explanatory mechanism for the two-way association between demands and burnout, was not included in these longitudinal studies. Future research could include three or more measurement waves in order to highlight the interplay between demands, burnout, and self-undermining over time, and to highlight the loss cycle.

## 5. Conclusions

During the COVID-19 pandemic, healthcare workers reported high levels of burnout. The prevalence of burnout was high, most likely due to increased job demands, such as the risk of infection, work pressure, making life-or-death prioritizing decisions, and a heavy workload. The results of the present study suggest that the impact of job demands during the pandemic could be underestimated, as these demands could generate a loss cycle (a high level of burnout due to demands caused by the outbreak can lead to self-undermining behaviors such as poor communication, errors, and interpersonal conflicts, which further increase demands). In order to avoid loss cycles during crises, health organizations could implement psychological interventions to reduce workers’ burnout, such as relaxation techniques, cognitive behavioral therapy, work-related knowledge and skills development, and job crafting interventions.

## Figures and Tables

**Table 1:** Means, standard deviations, reliabilities, and correlations between study variables (*N* = 523).

	*M*	SD	*α*	1	2	3	4
1. Work–family conflict	3.71	1.63	0.93				
2. Lack of comfort with working conditions	2.79	0.84	0.76	0.14 **			
3. Lack of equipment and supplies	2.57	0.92	0.75	0.33 ***	0.23 ***		
4. Burnout	1.35	0.93	0.89	0.30 ***	0.22 ***	0.28 ***	
5. Self-undermining	1.44	0.42	0.74	0.25 ***	−0.05	0.21 ***	0.45 ***

*Note: M* = mean; SD = standard deviation; *α* = Cronbach’s alpha; ** *p* < 0.01; *** *p* < 0.01.

## Data Availability

The data presented in this study are available on request from the corresponding author. The data are not publicly available due to confidentiality reasons.
